# Impact of Digital Media, School Problems, and Lifestyle Factors on Youth Psychosomatic Health: A Cross-Sectional Survey

**DOI:** 10.3390/children11070795

**Published:** 2024-06-28

**Authors:** Verena Barbieri, Giuliano Piccoliori, Adolf Engl, Christian J. Wiedermann

**Affiliations:** 1 Institute of General Practice and Public Health, Claudiana College of Health Professions, 39100 Bolzano, Italychristian.wiedermann@am-mg.claudiana.bz.it (C.J.W.); 2 Department of Public Health, Medical Decision Making and Health Technology Assessment, University of Health Sciences, Medical Informatics and Technology, 6060 Hall, Tyrol, Austria

**Keywords:** psychosomatic complaints, HBSC, children and adolescents, screen time, school, family, COVID-19, health status, gender

## Abstract

Background: Post-pandemic psychosomatic complaints in children and adolescents have been underreported. This study investigated psychosomatic complaints in children and adolescents in Northern Italy in 2023, with the aim of identifying changes in predictors and vulnerable subgroups. Methods: Cross-sectional data representative of scholars from a northern Italian province were analyzed using the Health Behavior in School-aged Children (HBSC) checklist. The sum scores, count data, and dichotomized data were examined, and predictor effects were expressed using a linear regression model for the sum scores. Results: Data from 4525 participants (aged 7–19 years, 50.5% girls) were analyzed. Psychosomatic symptoms remained unchanged after the pandemic. Significant gender differences were noted, especially in older age groups, with girls reporting more complaints. Factors such as children’s health, digital media use, and school problems significantly influence psychosomatic outcomes. While migration background negatively affected girls’ psychosomatic well-being, boys showed improvement through sports. Conclusion: The psychosomatic well-being of children and adolescents did not improve after the pandemic. These findings indicate the need for targeted interventions, improved health literacy, and ongoing monitoring to support the mental well-being of this vulnerable population. Reducing screen time remains a critical strategy for enhancing youth well-being.

## 1. Introduction

Psychosomatic complaints encompassing a range of physical symptoms without a clear medical cause [1] have been a focus of concern among children and adolescents. The Health Behavioral in School-aged Children Symptom Check List (HBSC-SCL) is a widely recognized international tool used for over four decades to assess subjective, non-clinical health complaints across diverse populations [2,3]. Pre-pandemic studies across Europe consistently reported an upward trend in psychosomatic complaints among children and adolescents [4,5,6,7,8,9,10]. In Italy, from 2006 to 2018, there was a significant increase in these complaints, particularly among girls [11,12,13,14,15,16,17]. Similarly, research in Germany noted a rise in psychosomatic complaints from 2009 to 2022, correlating with age, and showing a higher prevalence among girls [18,19]. The coronavirus disease (COVID-19) pandemic has exacerbated these issues. Longitudinal studies in Germany documented a marked decline in psychosomatic well-being during the pandemic, with complaints persisting post-pandemic [20,21,22]. Ravens-Sieberer et al. [22] highlighted that psychosomatic complaints surged during the pandemic and continued to increase thereafter.

Several factors have been identified as predictors of psychosomatic symptoms. Gender differences were significant, with higher rates among girls. Studies between 2010 and 2018 illustrated a worsening trend in teenagers’ well-being, particularly among 15-year-old girls, influenced by school pressure and a lack of social support [13]. Other factors, such as age, family situation, socioeconomic status, and health behaviors, are also associated with psychosomatic complaints [9,10,11]. The relationship between psychosomatic and mental health problems and problematic Internet use and social media use has been noted in Slovakian and Italian studies, respectively [10,14]. Pre-pandemic increases in symptoms are also linked to nutritional behavior and physical activities [15,16,17]. Moreover, parents’ mental well-being during the pandemic has been shown to impact children’s psychosomatic health. In Italy, parental fear related to the pandemic adversely affects children’s mental well-being [23,24]. Therefore, assessing parental mental health in the post-pandemic period is crucial for understanding children’s psychosomatic well-being.

This study focuses on South Tyrol, a unique region in northern Italy bordering Austria, characterized by a blend of German- and Italian-speaking communities. This multicultural setting at the heart of Western Europe provides a distinctive backdrop for examining the psychosomatic health of young people. Surveys in 2021 and 2022 on ‘Corona and Psyche in South Tyrol (COP-S) indicated persistent mental health challenges among students [25,26], aligning with findings from an Austrian–South Tyrol investigation [27]. Previous studies have highlighted that extended digital media use, passive sports behavior, parental mental well-being, and the female gender negatively impact the mental well-being of children and adolescents.

Building on these findings, the third COP-S study in 2023 aimed to examine the post-pandemic period in detail. Specifically, this study focused on four key objectives, which can be translated into the following research questions:

1. What are the levels of psychosomatic complaints among children and adolescents after the resumption of normal life following the pandemic?
2. What are the associations and differences in psychosomatic complaints between the sexes, and how can gender-specific strategies be developed to reduce these complaints?
3. How are psychosomatic complaints related to the general health status of children and their parents, daily school activities, family and leisure time behavior, and general demographic factors?
4. What are the key challenges in the post-pandemic period that affect the well-being of adolescents, and how can these challenges be addressed to ensure their well-being?

Studies suggest that the easing of COVID-19 restrictions and a return to normalcy may not directly lead to a reduction in psychosomatic complaints, as ongoing pandemic-related challenges continue to impact well-being [28]. The present study aims to provide new perspectives on the evolution of psychosomatic problems among children and adolescents in Northern Italy in 2023, filling gaps in the literature and offering insights into vulnerable subgroups’ post-pandemic psychosomatic complaints. Understanding the specific impact of the COVID-19 pandemic in the regional context is essential. The findings will inform targeted interventions and policies to improve well-being, particularly in the face of post-pandemic challenges. Findings from the unique context of South Tyrol can be applied to other multicultural settings.

## 2. Methods

### 2.1. Study Design and Sample

**Figure 1:**
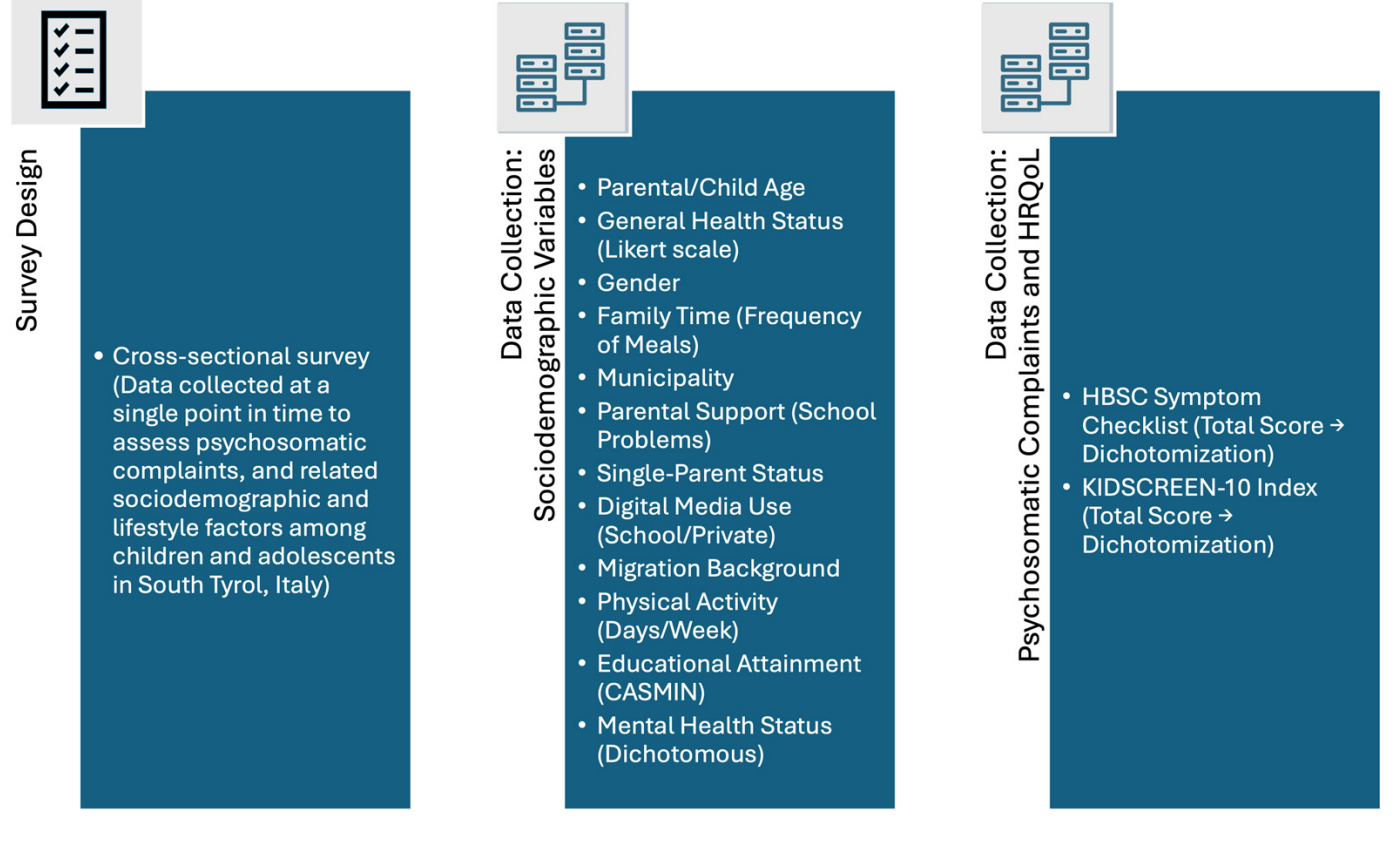
Methodological design and data collection of the cross-sectional survey in South Tyrol.

This study used a cross-sectional design to assess the psychosomatic well-being of children and adolescents in South Tyrol (Figure 1). Data were collected in April 2023 using the SoSci Survey Software, Version 3.2.46 (SoSci Survey GmbH, Munich, Germany). The questionnaire was primarily based on the German ‘Corona and Psyche’ (COPSY) study [22], allowing a comparable approach to accurately reflect the evolving nature of the impact of the pandemic on adolescents’ well-being.

The study sample consisted of students aged 7–19 years who were enrolled in public schools in the province. A link to the online questionnaire was distributed to all families through public school directories. Parents or guardians completed proxy questionnaires for children aged 7–10 years. Adolescents aged 11–19 years were given the opportunity to complete self-report forms in addition to parental (proxy) questionnaires, providing a dual perspective to South Tyrol data collection [25,26]. The sampling method accurately represented the age and gender demographics of the South Tyrolean student population, as reflected in regional statistics and previously described [25]. The sampling method established in the 2021 and 2022 surveys was repeated in the 2023 survey, with minor optimizations regarding the invitation reminders.

The response rate for the survey was 23%, with approximately 75% of the responses being analyzable after data cleaning.

### 2.2. Measures

#### 2.2.1. Sociodemographic Variables, General Health Status and Pandemic-Related Burden

Sociodemographic variables included parental and child age, gender, and municipality, as well as parental single-parent status, migration background, and educational attainment based on the Comparative Analysis of Social Mobility in Industrial Nations (CASMIN) index [29,30]. The mental health status of parents and children was assessed using the dichotomous question: “Do you/your child currently have a mental disorder diagnosed by a doctor, psychologist, or other professional?”

The survey included both proxy- and self-report versions covering various aspects of children’s current health status using a 5-point Likert scale ranging from 1 indicating “excellent” to 5 indicating “poor.” Family time was measured using the frequency of family meals in the past week (5-point Likert scale ranging from 1 indicating “never” to 5 indicating “every day”). The amount of time parents spent helping their children with school problems was also assessed (5-point Likert scale from 1 indicating “never” to 5 indicating “always”).

Digital media use was assessed both for school-related purposes (7-point Likert scale from 1 indicating “never” to 7 indicating “5 h or more”) and for private purposes (7-point Likert scale from 1 indicating “never” to 7 indicating “5 h or more”). In addition, the number of days per week with more than one hour of physical activity was recorded (ranging from 1 indicating “0 days” to 8 indicating “7 days”). These questions were derived from the Corona and Psyche (COPSY) Germany 2020 questionnaire [20,21,22].

#### 2.2.2. Psychosomatic Complaints and Health-Related Quality of Life

As in German COPSY studies [20,21,22], the following instruments were used:

Health Behavior in School-aged Children Symptom Checklist (HBSC): This checklist identifies psychosomatic complaints with items such as, “How often has your child had headaches in the past week?” Eight psychosomatic problems were assessed: headaches, stomachaches, backaches, feeling down, irritability, feeling nervous, sleep problems, and dizziness. Parents and adolescents were asked. Responses were recorded on a 5-point scale ranging from 1 indicating “daily” to 5 indicating “not at all” [31,32]. The total score was calculated when all eight questions were answered, with a higher score indicating better psychosomatic conditions (fewer complaints). The number of different complaints per week was assessed as count data, and psychosomatic complaints were dichotomized according to as 1 indicating “at least 3 psychosomatic complaints per week” and 0 indicating “less than 3 psychosomatic complaints per week.”

KIDSCREEN-10 Index: This index measures health-related quality of life (HRQoL) in children and adolescents and is a valid measure of a common HRQoL factor in this population. An example item is “Has your child felt fit and well?” The ten questions cover physical, psychological, social, and school-related items and are presented on a 5-point scale [33] with response values ranging from 1 indicating “not at all” to 5 indicating “extremely.” According to international norms, responses were summed into a total score and then dichotomized into 1 indicating “low” and 0 indicating “normal/high” HRQoL.

### 2.3. Data Analysis

Data were analyzed separately for two age groups: 7–10 and 11–19 years. When necessary, ordinal data were aggregated into dichotomous data, with documentation provided for these transformations. Cronbach’s alpha was calculated for the HBSC-SCL and HRQoL scales to assess their reliability.

Descriptive statistics were reported as mean ± standard deviation (SD) for metric variables, median and interquartile range ([1st quartile; 3rd quartile]) for count data, and absolute numbers and percentages for nominal data. Nominal data were compared using chi-square tests for independent samples and McNemar’s tests for paired samples to compare proxy- and self-reported data. The Mann–Whitney U test was used to compare ordinal and metric data between independent groups, while the Wilcoxon rank-sum test was used for paired data. Spearman’s correlation coefficient was calculated for correlations with the count data. The Bonferroni correction was applied to account for multiple tests.

The two key outcomes—proxy- and self-reported psychosomatic complaints—were analyzed as dichotomous variables and as sum scores based on both parents’ and adolescents’ questionnaires. For the sum scores, a linear regression model was used, accounting for demographic, health-related, and current lifestyle predictors. Pearson’s correlation coefficients were calculated for all predictors. Regression diagnostics were conducted to check for independence and normality of residuals, homoscedasticity, multicollinearity (Variance Inflation Factor, VIF), and autocorrelation of error terms (Durbin–Watson statistic). Outliers were identified using Cook’s distance and centered leverage points.

The sample size for the linear regression model was calculated using G-Power version 3.1. To detect a small-medium effect size (R^2^ 0.05) with a type I error probability of 0.05 and a power of 0.95 with 14 predictors, a sample size of 529 was required.

All statistical analyses were performed using the IBM SPSS Statistics for Windows (version 25.0; IBM Corp., Armonk, NY, USA).

## 3. Results

### 3.1. Levels of Psychosomatic Complaints after the Resumption of Normal Life

#### 3.1.1. General Overview

In total, 6044 parents participated in the survey, with 74.9% of the questionnaires deemed valid for analysis. The number of self-reports received from adolescents was 1831, representing 60.2% of 3037 participants in this age group.

Cronbach’s alpha for self-reported psychosomatic complaints was about 0.84, while for proxy-reported psychosomatic complaints it was about 0.82. For proxy-reported HRQoL, Cronbach’s alpha was approximately 0.86, and for self-reported HRQoL it was approximately 0.87.

Baseline characteristics such as age, gender, migration background, and single parenthood of the adolescents were found to be representative of the general South Tyrolean population, according to data from the Provincial Institute of Statistics of Bolzano. Detailed descriptive statistics are presented in Table 1.

While age, gender, and municipality were mandatory for parents and children in the questionnaire, some proxy reports had missing data: 2.4% for single parenthood, 6.2% for migration background, 4.1% for parental educational level, 8.9% for parental mental health problems, and for children’s mental health problems. Additional missing data in proxy-reported variables included 11.3% for actual days of sports, 12.9% for actual screen time hours for school concerns, 11.6% for actual screen time hours for private concerns, 16.5% for the actual general child’s health status, 11.0% for days of family meals together, and 12.6% for frequency of parental help with school problems. In self-reports, missing data were noted for days of sports (11.4%), screen time hours for school concerns (14.2%), screen time hours for private concerns (14.6%), actual child’s health status (11.6%), days of family meals together (12.1%), and frequency of parental help with school problems (5.2%). The percentages in Table 1 refer to available data.

McNemar’s test for dichotomized variables (last column) revealed significant differences between proxy- and self-reported data for variables related to leisure time behavior. Adolescents reported significantly better perceptions of their exercise behavior than their parents, and they reported more frequent use of digital media for personal and school purposes than their parents. In addition, adolescents reported more family meals and more parental help with school problems than their parents did. No significant differences were found between the proxy- and self-reported general health and low HRQoL among the adolescents.

#### 3.1.2. Gender Differences

**Figure 2:**
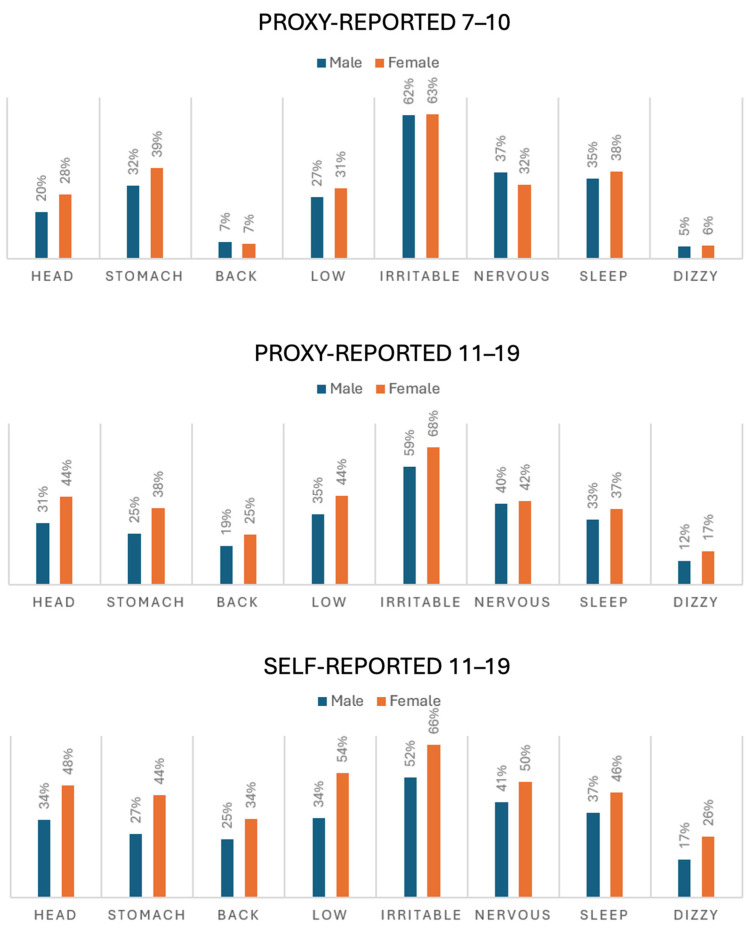
Percentages of psychosomatic complaints reported by gender and age group. Head, stomach, and back refer to aches; low, irritable, nervous, and dizzy refer to feelings; sleep refers to difficulties related to sleep.

The percentages of reporting the eight psychosomatic complaints at least once a week are detailed in Figure 2 separately for males and females. To account for multiple testing, only significant values after the Bonferroni correction were reported. The Wilcoxon rank test was used to compare the paired answers of the adolescents and their parents.

For headaches, stomachaches, backaches, sleeping difficulties, and dizziness, both genders reported significantly fewer psychosomatic complaints in proxy reports than in self-reports. After adjusting for multiple testing, no significant difference was found between proxy- and self-reports for irritability in either sex. However, for females, proxy reports indicated significantly fewer feelings of being low and nervous than self-reports.

In self-reports, girls reported significantly more psychosomatic complaints than boys for all eight types of complaints. In proxy reports for the younger age group (7–10 years), girls showed significantly more complaints concerning headaches (*p* = 0.001) and stomachaches (*p* = 0.003) after adjusting for multiple testing, while no significant gender differences were observed for other complaints. In the adolescent group (11–19 years), parents reported significantly more complaints from girls regarding headaches (*p* < 0.001), stomachaches (*p* < 0.001), backaches (*p* = 0.001), feeling low (*p* < 0.001), and dizziness (*p* = 0.001). Complaints of feeling nervous and sleeping difficulties did not show significant gender differences after the Bonferroni correction.

Table 2 provides an analysis of the overall HBSC sum score, number of psychosomatic complaints per week, and percentage of individuals experiencing at least three psychosomatic complaints per week for both genders.

##### Comparison of Results with 2021 and 2022 Data

The results for 2023 were compared with the original data published in the 2021 and 2022 surveys [25,26]. For self-reports, no significant changes were observed over the years for either boys or girls.

In the younger age group (7–10 years), proxy reports revealed a significant increase in stomachaches over the years, with rates rising from 27.5% in 2021 to 31.1% in 2022 and 35.4% in 2023 (*p* < 0.001). There was also a significant decrease in complaints about irritability in 2022 compared with 2021 and 2023, with a rate of 61.2% in 2021, dropping to 56.6% in 2022, and rising again to 62.5% in 2023 (*p* = 0.002). Additionally, there was a significant decrease in sleeping difficulties in 2022 compared to 2021 and 2023, with rates of 36.5% in 2021, 29.9% in 2022, and 36.2% in 2023 (*p* < 0.001).

For the older age group (11–19 years), proxy reports showed a significant increase in headaches, with rates going from 33.9% in 2021 to 39.2% in 2022 and slightly decreasing to 37.1% in 2023 (*p* < 0.001). There was also a significant decrease in irritability from 2021, with rates of 67.1% in 2021, decreasing to 62.6% in 2022, and slightly increasing to 63.3% in 2023 (*p* < 0.001).

All other psychosomatic complaints did not show significant changes over the years.

#### 3.1.3. Age Differences

Detailed analyses of associations with age showed a positive correlation for self-reported complaints in males for backaches (0.101, *p* < 0.01) and feeling low (0.099, *p* < 0.01). For females, there was a positive correlation with age for headaches (0.210, *p* < 0.001), stomach (0.102, *p* < 0.01), backaches (0.193, *p* < 0.001), feeling low (0.298, *p* < 0.001), irritability (0.154, *p* < 0.001), feeling nervous (0.151, *p* < 0.001), and dizziness (0.179, *p* < 0.001). No association was found for sleep problems among girls.

For proxy-reported data for boys, a positive correlation with age was found for headaches (0.115, *p* < 0.001), backaches (0.202, *p* < 0.001), feeling low (0.103, *p* < 0.001), and feeling dizzy (0.121, *p* < 0.01), whereas a negative correlation was found for stomachaches (−0.088, *p* < 0.001) and irritability (−0.048, *p* < 0.05). For proxy-reported complaints in girls, there was a positive association between age and headaches (0.207, *p* < 0.001), backaches (0.276, *p* < 0.001), feeling low (0.230, *p* < 0.001), irritability (0.100, *p* < 0.001), feeling nervous (0.182, *p* < 0.001), and dizziness (0.188, *p* < 0.001).

**Figure 3:**
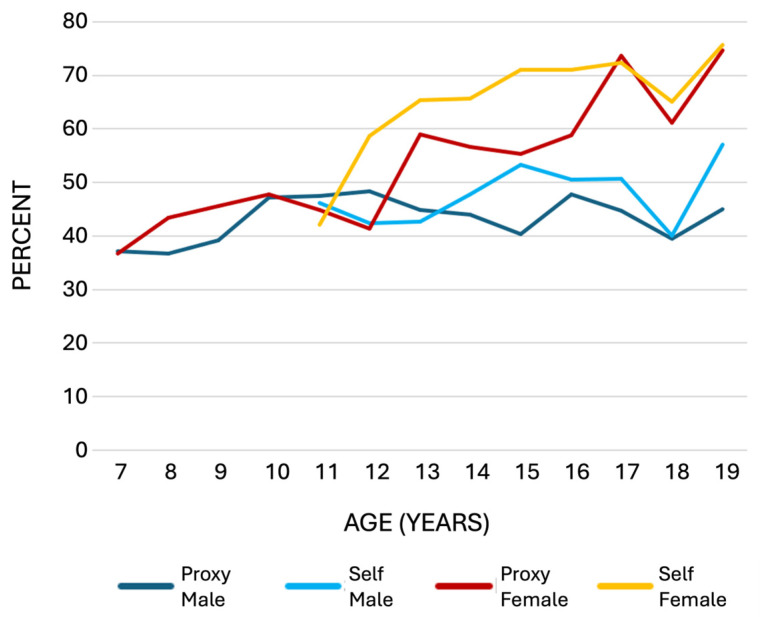
Percentages of proxy- and self-reported cases with at least three psychosomatic complaints per week, categorized by age and gender.

Age differences for the dichotomous variable “at least 3 psychosomatic complaints a week” are shown for boys and girls in Figure 3. As seen in Table 2, both self- and proxy-reported data revealed significant gender disparities in the age group of 11–19 years but not in proxy reports of children aged 7–10 years. Girls reported more psychosomatic complaints than boys did. Simultaneously, age was found to play a role, albeit inconsistently, in the number of reported psychosomatic complaints. In the older age group, the overall HBSC sum score for girls (−0.217, *p* < 0.001) and their parents (−0.201, *p* < 0.001) correlated significantly with age, whereas no correlation was found in the younger age group. The proxy- and self-reported psychosomatic complaints of boys did not show any age-related changes in either age group.

### 3.2. Associations between Psychosomatic Complaints and General Health Status, Daily Activities, and Demographic Factors

Associations of psychosomatic complaints with health-related, demographic, and lifestyle factors were investigated for proxy- and self-reported data in two age groups: 7–10 years and 11–19 years.

#### 3.2.1. Association of Proxy- and Self-Reported HBSC Sum Scores with Health-Related Predictors

Self-reported higher HBSC scores were associated with dichotomous variables of low HRQoL (−0.544, *p* < 0.001) and very good/excellent self-perceived health status (0.429, *p* < 0.001). In both the younger and older age groups, correlations with the proxy perceived low HRQoL (−0.471, *p* < 0.001) and good/excellent health status (0.359 and 0.453, respectively, *p* < 0.001) were similar.

Proxy-reported higher HBSC scores were negatively associated with children’s mental health problems (−0.208, *p* < 0.001) and parents’ mental health problems (−0.111, *p* < 0.001) in the younger age group. In the older age group, mental health problems (−0.348, *p* < 0.001) and parental mental health problems (−0.113, *p* < 0.001) were also negatively associated with proxy-reported higher HBSC scores. For self-reports, children’s mental health problems (−0.302, *p* < 0.001) were negatively associated with higher HBSC scores, whereas no association was found with parental mental health problems. Low HRQoL and very good/excellent health status were associated with self-reports (−0.429, *p* < 0.001) as well as proxy-reports in both age groups (−0.381 and −0.475, respectively, *p* < 0.001).

Parental mental health problems were significantly positively associated with children’s mental health problems in the younger (0.113, *p* < 0.001) and older age groups (0.117, *p* < 0.001), positively associated with parent-reported low HRQoL in the younger (0.140, *p* < 0.001) and older (0.086, *p* < 0.001) age groups, and negatively associated with proxy-reported very good/excellent health status of the children in the younger (0.064, *p* < 0.05) and older (0.087, *p* < 0.01) age groups. No association was found between self-reported low HRQoL and very good or excellent health status.

Mental health problems were positively associated with proxy-reported low HRQoL (0.172, *p* < 0.001), negatively associated with very good/excellent health status (−0.191, *p* < 0.001) in the younger age group and in the older age group with proxy-reported (0.312, *p* < 0.001) and self-reported (0.292, *p* < 0.001) low HRQoL, and negatively associated with self-reported (−0.239, *p* < 0.001) and proxy-reported (−0.299, *p* < 0.001) very good/excellent general health status.

In the linear regression model, general health status and children’s and parental mental health were included. Low HRQoL was not included because of its correlation with the other health-related variables. Age and sex were only slightly associated with health-related predictors (<0.2 each). Generally, all associations were slightly stronger in the older age group.

#### 3.2.2. Demographic Predictors for Proxy- and Self-Reported HBSC Scores

Proxy-reported higher HBSC scores were not associated with single parenthood, migration background, urban residency, or low parental education in the younger age groups. In the older age group, proxy-reported higher HBSC scores were slightly negatively associated with single parenthood (−0.065, *p* < 0.01), migration background (−0.051, *p* < 0.05), and urban residency (−0.066, *p* < 0.01) but not with low parental education.

Higher self-reported HBSC scores were negatively associated with single parenthood (−0.061, *p* < 0.05), migration background (−0.083, *p* < 0.01), and urban residency (−0.072, *p* < 0.01), but not with parental educational level. Demographic predictors were not or only slightly (<0.2) associated with each other and health-related predictors.

#### 3.2.3. Lifestyle Predictors for Proxy- and Self-Reported HBSC Scores

Self-reported HBSC scores were positively associated with parental help with school problems (0.166, *p* < 0.001), family meals together (0.146, *p* < 0.001), and the number of days with more than 60 min of sports (0.209, *p* < 0.001), and negatively associated with the use of digital media for school (−0.133, *p* < 0.001) and private concerns (−0.236, *p* < 0.001). Predictors were only slightly associated with each other and with demographic and health factors (<0.25 each). Age was strongly associated with the use of digital media for school concerns (0.430, *p* < 0.001).

In the younger age group, proxy-reported HBSC scores were positively associated with parental help with school problems (0.093, *p* < 0.01), family meals together (0.161, *p* < 0.001), and the number of days with 60 min of sports or more (0.162, *p* < 0.001), and negatively associated with hours of digital media use for school concerns (−0.140, *p* < 0.001) and for private concerns (−0.158, *p* < 0.001). Predictors were only slightly associated with each other and with demographic and health factors (<0.25 each).

In the older age group, proxy-reported HBSC scores were positively associated with parental help with school problems (0.114, *p* < 0.001), family meals together (0.161, *p* < 0.001), and the number of days with more than 60 min of sports per week (0.024, *p* < 0.001), and negatively associated with hours of digital media use for school (−0.154, *p* < 0.001) and private (−0.252, *p* < 0.001) concerns. Predictors were only slightly associated with each other and with demographic and health factors (<0.25 each). Only age was strongly associated with digital media use for school concerns (0.471, *p* < 0.001), indicating that age should be carefully considered in the linear regression model.

#### 3.2.4. Linear Regression Model to Predict Proxy- and Self-Reported HBSC Sum Scores

The findings indicate a significant association between female sex and an increase in psychosomatic complaints, particularly with advancing age in the older age group. Consequently, in the regression model analysis, the interaction between age and female gender was included (Table 3).

In the linear regression model for the proxy-reported HBSC sum score in the younger age group, no demographic variables were significant, while all health factors had a significant effect. Parental and children’s mental health problems had a negative effect, while very good/excellent health status had a positive effect. In contrast, for the older age group, sports activities had a significant positive effect on the HBSC sum score, whereas digital media use for private concerns had no effect. Extended parental help with school problems and digital media use for school concerns had a significant negative effect on proxy-reported good psychosomatic conditions in both age groups. In the older age group, proxy-reported good psychosomatic status was negatively associated with female gender with increasing age (female age), urban residency, and parental and children’s mental health problems. Very good or excellent health status had a positive effect. None of the other demographic variables were significant.

Self-reported better psychosomatic conditions were negatively associated with female gender with increasing age (Age × Female), urban residency, and children’s mental health problems but not with parental mental health problems. A positive effect of the actual health status was found. Lifestyle predictors had partly significant effects: intensive use of digital media for private concerns had a negative effect, whereas use for school concerns had no effect. Parental help with school problems had a positive effect, the opposite of what was found in the proxy reports. No effect on sports behavior was observed.

The corrected R^2^ was higher for the older age group, indicating that the sum score of psychosomatic complaints in this group was more likely to be explained by the predictors used. Regression diagnostics of proxy-reported sum scores showed a Durbin–Watson statistic of 1.91 in the younger and 0.96 in the older age group, and 0.91 for the self-reported sum score. All variance inflation factor (VIF) values were lower than 1.2. Residuals were centered around 0 with randomly distributed variance, and the normal PP-Plot showed that residuals clustered around the line. Cook’s distance and centered leverage point analyses identified some outliers; however, recalculation of the models excluding these outliers confirmed the initial results.

Recalculation of the linear regression models for the older age group by gender showed that in self-reports, beyond general health status, children’s mental health problems, private use of digital media, and parental help with school problems, there was a significant negative effect of living in an urban area and a significant positive effect of playing sports for males. For females, in addition to the aforementioned parameters, there was a significant negative effect of older age and migration background. The adjusted R^2^ values were 0.191 for boys and 0.321 for girls.

For proxy reports, for both genders, the variables general health status, children’s mental health problems, more hours of digital media use for private and school concerns, and family meals together were predictive factors. Additionally, migration background had a significant negative effect on girls’ psychosomatic complaints. For boys, age was positively associated with fewer psychosomatic complaints, whereas parental mental health problems, parental help with school problems, and living in an urban area were negatively associated with fewer psychosomatic complaints. The adjusted R^2^ values were 0.265 for boys and 0.329 for girls.

## 4. Discussion

This study aimed to assess the psychosomatic well-being of children and adolescents in South Tyrol in 2023 using self-reports from adolescents and proxy-reports from parents. With a high participation rate, the results provide an overview of psychosomatic complaints and their association with demographic, health-related, and lifestyle factors. The reliability of the measures was confirmed by Cronbach’s alpha values, which indicated high internal consistency for both self-reported and proxy-reported psychosomatic complaints and HRQoL.

The survey revealed that girls reported significantly higher levels of psychosomatic complaints than boys, particularly as they aged, whereas boys showed improved psychosomatic well-being with age. Additionally, extensive digital media use for private concerns emerged as a significant predictor of psychosomatic complaints for both genders. The associations between lifestyle factors and psychosomatic health highlight the significant roles of digital media use, physical activity, and family dynamics. The negative impact of extensive digital media use on psychosomatic complaints contrasts with the positive effects of physical activity and family meals, aligning with existing research that advocates a balanced lifestyle to enhance adolescent well-being [34,35,36,37].

### 4.1. Analysis of Psychosomatic Complaints Post-Pandemic in Youth

A comparative analysis with data from the 2021 and 2022 surveys showed some temporal changes, especially in proxy reports. During the post-pandemic period, there were no significant changes in psychosomatic complaints among children and adolescents between 2021 and 2023. This lack of improvement is particularly relevant given that an increase in psychosomatic complaints compared to pre-pandemic levels has been documented due to factors such as lockdowns, quarantines, and social isolation [22,26,38]. The increase in abdominal pain and fluctuations in irritability and sleep disturbance over the years suggest evolving patterns in psychosomatic health that may be influenced by external factors, such as post-pandemic adaptations.

In line with our findings, a recent study from Switzerland also reported a continuous increase in psychosomatic symptoms such as sleep problems, weakness, weariness, and headaches among young people from 2017 to 2022, suggesting that the decline in well-being started before the pandemic and highlighting the need to consider broader systemic factors [39]. Another study from Chile reported a continuous increase in psychosomatic symptoms such as sleep problems, weakness, weariness, and headaches among young people from 2017 to 2022, also suggesting that the decline in well-being began before the pandemic [40]. However, there is a relative lack of studies on psychosomatic complaints performed at the end of the 2023 pandemic, highlighting the need for continued monitoring and research in this area.

### 4.2. Gender-Specific Psychosomatic Complaint Patterns and Intervention Strategies

Gender differences were prominent, with girls reporting significantly more psychosomatic complaints than boys in both proxy and self-reports, particularly in the older age group. In addition, age-related differences highlighted that psychosomatic complaints tend to increase with age, especially in girls, which underscores the complexity of perceived psychosomatic problems among children, adolescents, and their parents, which is influenced by age and gender and not always perceived in the same way by children and their parents. In the linear regression model, after correcting for other factors, girls developed more psychosomatic complaints with increasing age, while boys’ psychosomatic well-being improved. These findings suggest that targeted, possibly gender-specific, interventions could be beneficial, especially for children.

Proxy reports for children aged 7–10 years did not show significant gender differences in psychosomatic complaints. Therefore, the focus shifts to adolescents aged 11–19 years. In this group, girls reported significantly higher levels of psychosomatic complaints for almost all eight symptoms assessed. In addition, girls experienced a greater age-related increase in psychosomatic complaints than boys, illustrating the increasing disparity between boys and girls in both proxy- and self-reported psychosomatic complaints as they age. These findings are consistent with previous studies [12,13,18] and highlight the need for gender-specific interventions, particularly during adolescence. These gender differences are most apparent in Figure 3, which illustrates the increasing disparity between boys and girls in both proxy- and self-reported psychosomatic complaints with age.

The high level of digital media use among children and adolescents is an ongoing concern. While digital media use for educational purposes affected psychosomatic complaints only in the younger age group, screen time for private purposes significantly affected psychosomatic complaints in both self-reported and proxy-reported data for adolescents, making it a significant predictor of mental health problems.

In terms of gender-related interventions, the focus is on the use of digital media, especially smartphones for social media among girls, as highlighted in previous studies [10,14,15]. The present data confirm the negative effects of digital media use for private concerns on psychosomatic health for both genders [41,42,43,44,45,46]. Future studies should distinguish between different forms of digital media use, such as problematic social media and Internet use. It is necessary to examine the specific contexts in which digital media use occurs, as the impact on psychosomatic health may vary depending on the type of activity and social environment, such as passive consumption of content versus active engagement or content creation [33]. In addition, exploring the interplay between digital media use and other lifestyle factors such as physical activity and family dynamics can provide a more comprehensive understanding of how these elements collectively influence mental health.

The importance of targeted improvement in health literacy should be the focus of further research, particularly with a special emphasis on gender differences [47,48]. In the present study, girls’ psychosomatic health did not significantly benefit from sports in the older age group, whereas boys showed a significant improvement. This is a noteworthy finding considering that previous research has indicated a strong link between physical activity and mental well-being across various mental health symptoms, especially among girls [49]. A Canadian study found that daily physical activity significantly reduced psychosomatic complaints from 44% in girls and 57% in boys [50]. However, Gerber and Pühse [51] found no significant association between physical activity and psychosomatic complaints or stress in adolescents, suggesting that other factors, such as self-esteem, might play a more critical role in moderating the relationship between stress and psychosomatic health. Therefore, although physical well-being is crucial for overall mental health, the psychosomatic benefits for girls require further investigation.

### 4.3. Influence of Health, Lifestyle, and Demographics on Psychosomatic Complaints

This study revealed the significant negative effect of elevated screen time use for school concerns on psychosomatic well-being in the younger age group. In addition, there was a negative association between increased parental support for school concerns and psychosomatic well-being in both proxy-reported age groups. Interestingly, self-reported data showed a positive effect of parental support, suggesting that adolescents may feel more secure knowing that their parents are involved in their school problems, whereas parents may view this support as burdensome and prefer that school problems be resolved at school.

The literature supports the critical role of school-related factors in psychosomatic complaints [42]. For example, positive perceptions of school performance have been shown to be associated with fewer health complaints [42]. Psychosocial school factors, including social support within the school environment and reduced school workload, are important for improving mental health in first-year secondary school students [41]. Teacher support has been found to be more strongly associated with adolescents’ mental and physical health than family or peer support [52]. In addition, school stress has been found to increase more for girls than for boys, explaining a significant proportion of the gender difference in symptoms [46]. This underscores that school stress is a major contributor to the increase in psychosomatic symptoms for girls, but only a minor contributor for boys.

These findings suggest the need for interventions that address school-related stress, increase social support in the school environment, and reduce the burden of schoolwork to reduce psychosomatic symptoms in adolescents.

In this study, higher proxy-reported psychosomatic complaints in children and adolescents were strongly associated with parental mental health problems, whereas self-reported data did not show a significant association. This finding underscores the importance of monitoring children themselves and relying on self-reports to accurately assess their health status [53]. The significant association between child psychosomatic complaints and parental mental health emphasizes the interdependence of family members’ psychological well-being. This finding is consistent with research indicating the reciprocal influence of parental and child mental health [54]. Therefore, these findings suggest the need to incorporate parental mental health assessments into child mental health interventions. By recognizing and addressing the interconnected nature of family mental health, interventions may be more effective and comprehensive, ultimately contributing to improved outcomes for both children and their parents.

Significant differences were found between proxy- and self-reports of psychosomatic complaints, with adolescents consistently reporting more complaints than their parents did. Physical symptoms, such as headaches, stomachaches, backaches, dizziness, and sleeping difficulties, were reported significantly more frequently by adolescents. Conversely, emotional symptoms such as feeling low and nervous were more often noted in proxy reports by parents. These findings suggest that relying solely on proxy reports may not fully capture the extent of adolescents’ psychosomatic symptoms, emphasizing the importance of considering self-reports to accurately assess their health status.

### 4.4. Addressing Post-Pandemic Well-Being Challenges in Vulnerable Adolescent

Girls with migrant backgrounds have emerged as a particularly vulnerable group. This finding is significant because there is limited evidence on the psychosomatic health of this specific subgroup. Previous research [55] has examined psychosomatic complaints among first- and second-generation migrant adolescents in different countries, showing associations, but also significant differences between countries. The Italian HBSC study [56] suggested that perceived high levels of support among immigrant adolescents may moderate the impact of environmental stressors. These findings are consistent with international guidelines, which suggest that public health professionals should develop school interventions that promote inclusiveness, foster a supportive school environment, and involve the families of immigrant youth. This approach aims to mitigate the adverse effects of environmental stressors and to improve the overall well-being of this vulnerable population.

### 4.5. Limitations

This study had several limitations. Its cross-sectional design limits its ability to infer causality between health issues and their determinants. The reliance on self-reported data introduces potential recall or social desirability bias, especially regarding sensitive topics, such as quality of life. There may also be sampling bias, as participants who responded to the survey may have had better health conditions. The study did not consider other potentially significant factors, such as nutritional behavior or extended family support. For younger children, the use of proxy reports from parents could introduce bias, as parents with mental health issues might perceive their children’s health as more negative.

## 5. Conclusions

Increases in psychosomatic complaints and high personal screen time use persisted after the pandemic. The present findings confirmed previously observed trends, such as the gender gap in psychosomatic complaints and the influence of age. In addition, new vulnerable groups were identified, particularly migrant girls, who require close monitoring. Boys can alleviate these problems through sport, although this effect was not observed for girls. The discrepancy between adolescent self-reports and parental proxy reports indicates the need to improve health literacy among adolescents and their parents. Integrating health literacy into the school curriculum could enable teachers to effectively teach and monitor health behaviors. In addition, addressing school-related stress and managing screen time are critical to improving adolescents’ psychosomatic well-being. A limitation of this study is its reliance on self-reported and proxy-reported data, which may be subject to bias. The cross-sectional nature of the study also limits the ability to infer causality between the pandemic and psychosomatic complaints. In addition, the study did not control for other potential confounding factors, such as socioeconomic status, that could influence the observed trends. Future research should focus on longitudinal studies to establish causal relationships between pandemic-related factors and psychosomatic complaints. There is a need for tailored interventions to support the well-being of vulnerable populations, especially as social conditions evolve. Emphasis should be placed on improving health literacy among children and adolescents, taking into account the specific needs of different gender and age groups, as well as other vulnerable groups, such as children with parents with mental health problems or those from migrant families.

## Figures and Tables

**Table 1:** Sociodemographic and pandemic-related characteristics of children aged 7–10 and adolescents aged 11–19 of the COP-S 2023 proxy- and self-reported samples.

Variable	Proxy-Report		Proxy-Report	Self-Report	*p*-Value *
	7–19 Years *n* = 4525	7–10 Years *n* = 1488	11–19 Years *n* = 3037	11–19 Years *n* = 1831	
	M (SD)				
Age children	12.6 (3.6)	8.4 (1.2)			
Age parents	45.3 (6.3)	41.3 (5.6)			
	%				
Female gender children	50.6	49.3	51.2		
Female gender parents	89.8	90.6	89.4		
Migration background	10.2	11.5	9.5		
Low parental education	23.7	19.2	26.0		
Single parenthood	8.7	5.4	10.3		
Urban residency	24.5	19.5	27.0		
Parental mental health problems	4.2	4.6	4.0		
Childrens’ mental health problems	7.0	3.8	8.6		
Actual very good/excellent health status of the child	76.4	83.4	73.0	71.6	n.s.
Low HRQoL	19.8	12.2	23.5	21.9	n.s.
Sports activities (3 days+)	66.5	79.2	60.1	67.5	<0.001
Advanced use of digital media for school (3 h+)	10.4	0.6	15.1	17.9	<0.001
Advanced use of digital media for personal use (3 h+)	31.3	7.2	43.2	49.1	<0.001
Parents helped with school problems (always/often)	60.3	68.4	56.3	70.1	<0.001
At least 5 days with family meals together	74.7	83.4	70.5	75.4	0.001

* Self vs. proxy report paired samples: McNemar’s test Abbreviations: M, mean; SD, standard deviation; HRQoL, health-related quality of life.

**Table 2:** Analysis of psychosomatic complaints by age group and gender for self- and proxy-reported data.

Outcome	Gender	Proxy-Report		Self-Report	*p*-Value ^c^
		7–10 Years *n* = 1204	11–19 Years *n* = 2413	11–19 Years *n* = 1548	
		M (SD)	M (SD)	M(SD)	Wilcoxon-Rank sum Test
Sum score (metric)	Total	36.86 (3.34)	35.94 (4.44)	35.24 (4.94)	<0.001
	Male	37.01 (3.15)	36.52 (3.92)	36.11 (4.46)	<0.001
	Female	36.71 (3.53)	35.36 (4.84)	34.38 (5.23)	<0.001
	*p*-value ^a^	n.s.	<0.001	<0.001	
		Median [1Q;3Q]	Median [1Q;3Q]	Median [1Q;3Q]	Wilcoxon-Rank sum Test
Number of different psychosomatic complaints a week (ordinal)	Total	2 [1;4]	3 [1;4]	3 [1;5]	<0.001
	Male	2 [1;3]	2 [1;4]	2 [1;4]	<0.001
	Female	2 [1;4]	3 [1;5]	3 [2;5]	<0.001
	*p*-value ^a^	n.s.	<0.001	<0.001	
		%	%	%	McNemar-Test
At least 3 psychosomatic complaints a week (dichotomous)	Total	41.5%	51.0%	55.8%	<0.001
	Male	40.1%	44.8%	47.4%	0.005
	Female	43.0%	57.1%	64.0%	<0.001
	*p*-value ^b^	n.s.	<0.001	<0.001	

^a^ Mann–Whitney U test, gender comparison; ^b^ Chi-Square Test, gender comparison; ^c^ paired samples, proxy vs. self-reports; n.s. not significant.

**Table 3:** Linear regression models for Health Behavior in School-aged Children Symptom Checklist (HBSC) scores.

	Predictors of HRQoL Scores in Different Age Groups and Reporting Methods		
	Proxy-Reported HBSC Score (7–10) *n* = 1076	Proxy-Reported HBSC Score (11–19) *n* = 2278	Self-Reported HBSC Score (11–19) *n* = 1405
Corrected R^2^	0.198	0.308	0.281
Predictor	Beta [95%]	Beta [95%]	Beta [95%]
Constant term	34.39 [32.64;36.13] ***	37.81 [36.35;39.27] ***	34.09 [32.75;35.43] ***
Age (years)		n.s.	n.s.
Female gender		n.s.	n.s.
Age × Female gender		−0.08 [−0.10;−0.06] ***	−0.10 [−0.13;−0.07] ***
Parental mental health problems	−1.11 [−1.93;−0.29] **	−1.05 [−1.88;−0.23] *	n.s.
Children’s mental health problems	−2.43 [−3.37;−1.50] ***	−3.40 [−3.98;−2.81] ***	−3.63 [−4.49;−2.77] ***
Actual general health status (less than very good)	2.75 [2.25;3.25] ***	3.32 [2.94;3.69] ***	3.55 [3.03;4.06] ***
Days of family meals together	0.41 [0.21;0.61] ***	0.27 [0.13;0.42] ***	n.s.
Parental help with school problems	−0.26 [−0.43;−0.09] **	−0.23 [−0.37;−0.09] **	0.57 [0.35;078] ***
Use of digital media for private issues (hours)	n.s.	−0.46 [−0.58;−0.34] ***	−0.55 [−0.72;−0.39] ***
Use of digital media for school issues (hours)	−0.35 [−0.62;−0.08] ***	−0.25 [−0.37;0.12] ***	n.s.
Number of days with of at least 60 min of sports activities	0.17 [0.07;0.27] **	n.s.	n.s.
Single Parenthood		n.s.	n.s.
Urban residency		−0.45 [−0.81;−0.12] **	−0.81 [−1.30;−0.31] **
Migration Background		n.s.	n.s.

Abbreviations: HRQoL, health related quality of life; HBSC, Health Behavior in School-aged Children Symptom Checklist; *** *p* < 0.001, ** *p* < 0.01; * *p* < 0.05; n.s., not significant.

## Data Availability

The data presented in this study are available from the corresponding author upon request.
